# From Sting to Strain: A Case Report of Wasp Sting-Induced Multiorgan Dysfunction Syndrome

**DOI:** 10.7759/cureus.106687

**Published:** 2026-04-08

**Authors:** Abin Thomas, Avin Shaji John, Vishnu C Unnikrishnan, Sreekrishnan TP, Gireesh Kumar, Ritvik Sajan

**Affiliations:** 1 Emergency Medicine, Amrita Institute of Medical Science, Kochi, IND; 2 Pharmacy Practice, Amrita School of Pharmacy, Kochi, IND; 3 Emergency Medicine, Amrita Hospital, Kochi, IND

**Keywords:** acute kidney injury, hepatic dysfunction, multiple organ dysfunction syndrome (mods), rhabdomylosis, wasp sting envenomation

## Abstract

Wasp stings are benign, generally associated with localized pain and allergic reactions, but in rare cases, they may trigger life-threatening systemic toxicity, culminating in multiorgan dysfunction syndrome (MODS). We present the case of a 23-year old female who developed MODS following multiple wasp stings, manifesting with rhabdomyolysis, acute kidney injury (AKI), hepatic dysfunction, and necrotic skin lesions. Laboratory evaluation showcased markedly elevated creatine kinase (CK), liver enzymes, and renal parameters. She was managed with Adrenaline, corticosteroids, aggressive intravenous fluids, and supportive hepatorenal measures, leading to complete recovery within a month. This case serves as a reminder that rapid intervention and fast-paced multidisciplinary management can mean the difference between complete recovery and a fatal outcome.

## Introduction

Wasp stings are a frequent occurrence in tropical and subtropical regions, where human activity overlaps with the habitats of these insects [1]. In most cases, stings cause localized pain, erythema, and swelling, or an allergic reaction that resolves with symptomatic treatment. However, serious systemic toxicity and organ dysfunction can be triggered by multiple stings or even a single sting in susceptible individuals [2]. Wasp venom can produce a plethora of profound systemic effects, including rhabdomyolysis, hemolysis, thrombolysis, acute kidney injury (AKI), hepatic necrosis, and neurologic complications. Acute kidney injury (AKI) is one of the most commonly documented complications, typically caused by rhabdomyolysis and toxin-driven tubular damage. Inflammatory cascades involving mitochondrial deoxyribonucleic acid (mtDNA) and the stimulator of interferon genes (STING) pathway further exacerbate renal injury [3]. The mainstay of emergency management involves prompt administration of adrenaline, antihistamines, corticosteroids, and aggressive intravenous fluid therapy to maintain renal perfusion and prevent pigment-induced nephropathy [4]. Supportive measures with vasopressors, analgesics, and respiratory support may also be used in severe cases, while N-acetylcysteine and hepatoprotective measures have been helpful in mitigating hepatic injury [5]. Despite these interventions, mortality remains significant, particularly among patients presenting with shock, oliguria, or severe systemic involvement, with reported mortality ranging approximately 15% to 25% in severe envenomation cases [6]. However, mortality remains significant in patients presenting with shock, oliguria, or high inflammatory indices despite interventions. Here, we report the case of a young female patient who developed rhabdomyolysis, acute kidney injury (AKI), and hepatic dysfunction following multiple wasp stings and presented to the emergency medicine department of a tertiary care hospital.

## Case presentation

A 23-year-old South Asian lady was presented to the emergency department at Amrita Institute of Medical Science, Kochi, India. She was admitted after sustaining multiple unprovoked wasp stings while walking along a roadside near vegetation and flowers. Her prior medical history review showed that she had no comorbidities and was not on any regular medications. Immediately after the stings, she collapsed to the ground with a brief loss of consciousness lasting for about one minute.

**Figure 1:**
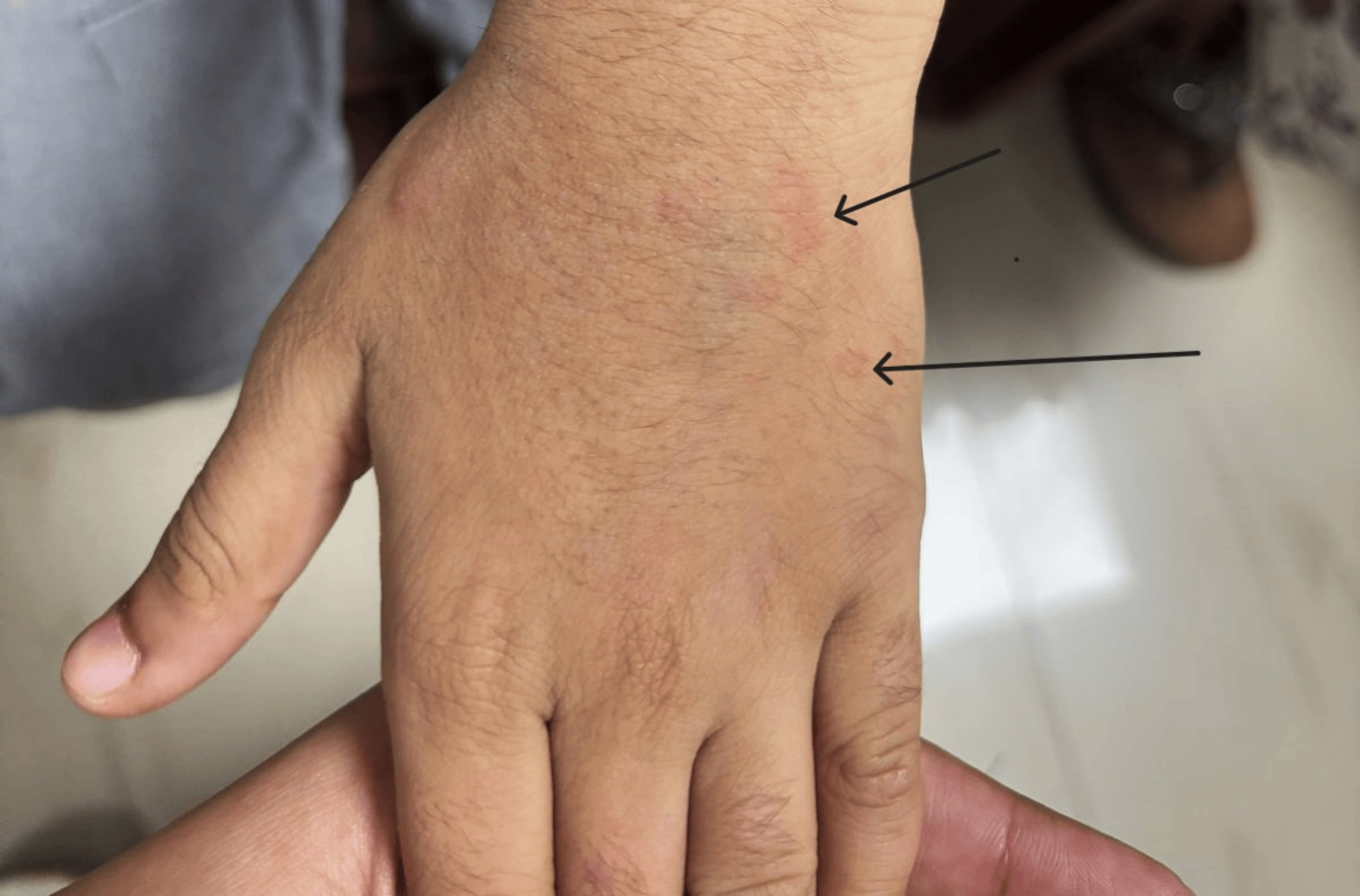
Clinical appearance of wasp-stung sites causing redness, erythema with localized vasculitis (arrows)

It was followed by an acute onset of breathlessness (modified Medical Research Council mMRC grade 4), severe pain, itching at sting sites, and generalized myalgia. She received initial symptomatic management at a local hospital and was referred to our tertiary care center for further management.

Clinical examination

Upon admission, the initial 10-second airway, breathing, circulation, disability, and exposure evaluation was performed and summarized in Table 1.

**Table 1:** Initial evaluation of patient SpO_2_: saturation of peripheral oxygen.

Parameters	Findings
Airway	Appears patent; no secretions
Breathing	Bilateral wheeze present; respiratory rate: 28 breaths/minute; SpO2: 95% on room air
Circulation	Blood Pressure: 90/50 mmHg: pulse rate: 106 beats/minute
Disability	Glasgow Coma Scale: 15 (eye response – 4, verbal response – 5, motor response – 6); pupils: 2.5 mm, bilaterally reacting
Exposure	Temperature: 98 °F; redness and edema over sting sites

Initial evaluation demonstrated abnormal vitals, including a blood pressure of 90/50 mmHg and a respiratory rate of 28 breaths/min, consistent with hypotension and tachypnea. Further examination of the skin showed multiple sting marks scattered over the body, many of which demonstrated central necrosis with surrounding erythema.

Investigations

Routine laboratory investigations revealed evidence of rhabdomyolysis, AKI, and hepatocellular injury, along with leukocytosis and myoglobinuria. This constellation of hepatic, renal, and muscular dysfunction, accompanied by systemic inflammation were indicative of multiorgan dysfunction (MODS) rather than isolated organ pathology. Detailed laboratory parameters are summarized in Table 2.

**Table 2:** Relevant laboratory findings of the patient. AST: aspartate aminotransferase, ALT: alanine aminotransferase, CK: creatine kinase, ABG: arterial blood gas, HCO₃⁻: bicarbonate, pCO₂: partial pressure of carbon dioxide, K⁺: potassium.

Laboratory Findings	Results	Reference Range
Serum urea	85.9 mg/dL	10-40 mg/dL
Serum creatinine	2.85 mg/dL	0.6-1.2 mg/dL
AST	2509.3 IU/L	0-40 IU/L
ALT	944.8 IU/L	0-40 IU/L
CK	67,300 U/L (peak 94,525 U/L at 8 hours)	<200 U/L
Urine myoglobin	+++	Negative
White cell count	18,100/µL	4000-11,000/µL
Arterial blood gas (ABG) pH	7.325	7.35-7.45
HCO₃⁻	16 mEq/L	22-28 mEq/L
pCO₂	37 mmHg	35-45 mmHg
K⁺	4.8 mmol/L	3.5-5.1 mmol/L

**Figure 2:**
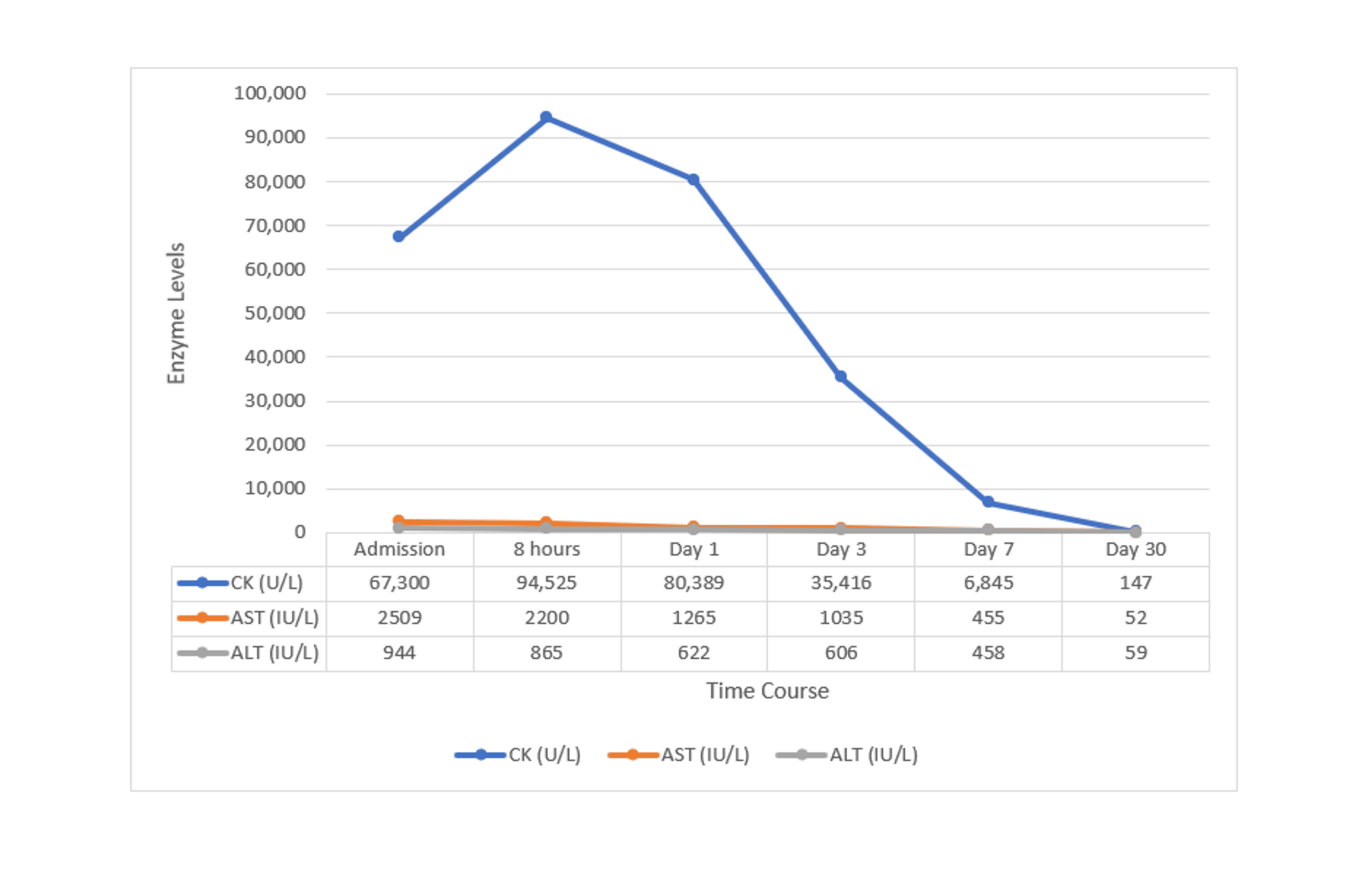
Serial trend of biochemical parameters. Trend of creatine kinase (CK), aspartate aminotransferase (AST), and alanine aminotransferase (ALT) levels are shown. CK levels peaked within the first eight hours, consistent with acute rhabdomyolysis, followed by a progressive decline. Liver enzymes (AST and ALT) also demonstrated gradual normalization, reflecting recovery from hepatocellular injury. The figure was created using Microsoft Word (Microsoft Corporation, Redmond, Washington).

Treatment

Upon admission, the patient was resuscitated with an intravenous bolus of Ringer's lactate (20 mL/kg), followed by maintenance infusion titrated to maintain urine output above 1 mL/kg/hour. Simultaneously, for management of anaphylaxis, intramuscular epinephrine 0.5 mg was administered into the anterolateral thigh, followed by intravenous pheniramine maleate 25 mg along with fluids. Intravenous hydrocortisone 100 mg was administered to reduce systemic inflammation and prevent late-phase anaphylaxis.

N-acetylcysteine (NAC) was initiated for acute liver injury using a standard intravenous protocol: a loading dose of 150 mg/kg over 1 hour, followed by 50 mg/kg over 4 hours, and then 100 mg/kg over 16 hours. The infusion was continued for a total of 48 hours based on clinical response.

In view of multiple stings and the risk of secondary bacterial infection, doxycycline 100 mg orally twice daily was initiated for prophylaxis. Supportive care included oxygen supplementation via face mask, analgesics for pain control (paracetamol 1 g intravenously as needed), and close monitoring of hepatic and renal function parameters throughout hospitalization.

The patient showed gradual clinical and biochemical improvement over the course of hospitalization and was discharged after a total length of stay of 10 days in stable condition. At discharge, renal and hepatic parameters had improved significantly compared to those at initial admission. The patient was advised regular outpatient follow-up, and at two-week follow-up, remained asymptomatic with near normalization of laboratory parameters.

## Discussion

Wasp venom is responsible for local and systemic symptoms arising from wasp stings, which can precipitate into multiorgan dysfunction if not promptly treated [6]. The venom constitutes a complex mixture of biological components, including phospholipase A1, hyaluronidase, mastoparan, melittin-like peptides, kinins, and biogenic amines such as histamine and serotonin [7]. These substances produce diverse effects: phospholipases and mastoparan disrupt cell membranes of blood cells, causing cytotoxicity and rhabdomyolysis; hyaluronidase facilitates the penetration of venom into tissues; and amines trigger pain, erythema, and inflammation [8]. Clinically, most patients develop local pain and erythema at the sting site, whereas a minority present with systemic manifestations, including anaphylaxis, hemolysis, AKI, hepatic dysfunction, and, in complex cases, progression to MODS [9]. Mortality has been strongly associated with multiple stings, delayed treatment, and systemic inflammatory responses [10].

In our patient, who sustained multiple unprovoked stings, clinical and laboratory data demonstrated a toxic systemic reaction, characterized by AKI, evident from markedly elevated CK levels and myoglobinuria. The incidence of AKI has been reported to be around 30 to 50% in wasp sting victims, according to recent studies [11]. AKI is mainly caused by the direct toxic effect of venom, secondary to rhabdomyolysis, which can progress to ischemic acute tubular injury and necrosis [12]. Management of rapidly deteriorating AKI involves renal replacement therapy such as hemodialysis or peritoneal dialysis [13]. Our patient was continuously hydrated with Ringer lactate solution to treat hypovolemia, which in turn helped improve renal parameters by the third day, without requiring renal replacement therapy. This likely occurred due to early hospitalization and aggressive hydration.

The incidence of hepatotoxicity following wasp stings is around 30% [14]. Identification of hepatic injury can be challenging, as elevation in transaminases can occur concurrently with rhabdomyolysis and hemolysis. Although a rise in serum AST (aspartate aminotransferase) and ALT (alanine aminotransferase) was found in our patient, it was accompanied by a parallel increase in CK, suggesting that muscle damage was the sole reason for the increase in liver enzymes. However, there were no cardiac or neurological complications in our patient. Intravenous infusion of NAC (N-acetylcysteine) was given for 48 hours in the patient. NAC infusion is the drug of choice for acetaminophen-induced acute liver failure [15]. Beyond this, NAC has demonstrated broader hepatoprotective effects through multiple mechanisms, including antioxidant activity, scavenging of free radicals, and improvement of hepatic microcirculation. It also enhances mitochondrial function and reduces oxidative stress-mediated cellular injury, which is especially relevant in toxin-induced and ischemic hepatic injury, such as seen in envenomation [16]. In cases of wasp sting-induced systemic toxicity, hepatic injury is often multifactorial, resulting from direct toxicity, hypoperfusion, rhabdomyolysis, and systemic inflammatory response. Although evidence is limited, recent studies suggest that NAC has been increasingly used as an off-label therapy in non-acetaminophen acute liver injury, where studies have shown improved transplant-free survival and better clinical outcomes, specifically in early stages of liver injury [17]. In our patient, NAC was initiated during the early phase of illness, which may have contributed to the favorable recovery and improvement in hepatic parameters.

The management of wasp stings remains supportive, with no specific antidote available. Thus, there are no established guidelines for the treatment of wasp envenomation. Anaphylaxis arising in severe cases must be promptly treated with epinephrine, as done in our case [18]. The addition of fluids and inopressors is crucial for maintaining organ perfusion pressure and preventing hypovolemia. Attention should be paid to preventing and treating electrolyte imbalances. Oral corticosteroid therapy can be initiated to prevent late-phase anaphylaxis, as done in our case, where the patient received a short course of injection hydrocortisone, which is also effective in mitigating systemic inflammation [19]. Early diagnosis and management were crucial, including removal of wasp stings and prompt correction of hypotension with intravenous fluids to preserve urine output. A recent case series on multiple wasp stings concluded that 89% of patients had leukocytosis. Leukocytosis is therefore a common occurrence in wasp sting cases and may not always signify infection [20]. This was also apparent in our case, and doxycycline was administered judiciously.

Recent studies have further highlighted the variability in clinical presentation and outcomes following wasp envenomation, emphasizing the importance of early risk stratification. Large cohort analyses have demonstrated that the severity of organ injury correlates with venom load and host inflammatory response, with biomarkers such as procalcitonin and creatine kinase serving as predictors of AKI and systemic complications [21]. Additionally, severe cases may require intensive care support, underscoring the role of critical care management in improving survival. Rhabdomyolysis remains a key mechanism contributing to renal injury, with toxin-mediated muscle breakdown leading to significant morbidity [22]. From a broader perspective, epidemiological studies indicate that delayed access to healthcare and lack of awareness contribute significantly to adverse outcomes, especially in tropical regions [23].

The novelty of this report lies in the early presentation and proactive management of patients with multiple wasp stings. Recognizing the grave risk associated with intravascular hemolysis, rhabdomyolysis, and AKI, early aggressive hydration and urine alkalinization were initiated to prevent further systemic complications. This timely intervention likely reduced the severity of AKI, minimized morbidity, shortened hospital stay, and avoided the need for dialysis.

## Conclusions

Wasp stings, though often considered minor, can lead to severe systemic toxicity and multiorgan dysfunction if not identified and managed promptly. This case underscores the importance of early recognition, aggressive fluid resuscitation, and multidisciplinary supportive therapy in preventing life-threatening complications such as rhabdomyolysis, acute kidney injury, and hepatic dysfunction. Administration of norepinephrine, corticosteroids, and N-acetylcysteine, along with careful monitoring of renal and hepatic parameters, contributed to the patient’s full recovery. Given the potential for rapid deterioration, clinicians, particularly in endemic regions, must maintain a high index of suspicion and initiate timely, evidence-based management to ensure favorable outcomes. Public awareness and preventive strategies should be adopted, as wasp stings have no specific antivenom. Treatment requires prompt hospitalization for aggressive hydration and urine alkalinization to prevent AKI secondary to rhabdomyolysis.
